# Development of multiplex RT-PCR assays containing an internal amplification control for the detection of dicistro-, iflaviruses and CBPV in honey bees. Part 1 - assays design and optimization

**DOI:** 10.1007/s11259-025-11012-3

**Published:** 2026-01-15

**Authors:** Dagmara Zdańska, Artur Rzeżutka, Krystyna Pohorecka

**Affiliations:** 1 Department of Parasitology and Invasive Diseases, Bee Diseases and Aquatic Animal Diseases, National Veterinary Research Institute, Al. Partyzantów 57, Puławy, 24-100 Poland; 2 Department of Microbiology of Food and Feed, National Veterinary Research Institute, Al. Partyzantów 57, Puławy, 24-100 Poland; 3 National Veterinary Research Institute, Al. Partyzantów 57, Puławy, 24-100 Poland

**Keywords:** Honey bee, Viruses, Multiplex RT-PCR, Internal amplification control, Detection

## Abstract

Viral infections can cause increased mortality of honey bee colonies. Because of an asymptomatic course of infections their detection in honey bees is only possible by the use of sensitive and specific diagnostic methods. Nowadays, the conventional RT-PCR assays are routinely employed for detection of honey bee viruses, but none of them have had their diagnostic usefulness confirmed through documented optimization and validation process. The aim of this study was to develop of the two in-house multiplex RT-PCR (mRT-PCR) assays for the detection of CBPV, iflaviruses (DWV-A, SBV) and dicistroviruses (ABPV, IAPV, BQCV) of bees. The sequences of the primers used in the developed Ifla-CBPV and Dicistro mRT-PCRs were refined to include point mutations observed in the respective genome fragments of honey bee viruses detected worldwide so far, including newly characterised virus strains from Poland. The optimization of the PCR mixtures composition and temperature-time profiles of the assays was performed. To avoid false negative results, an internal amplification control (IAC) was prepared and incorporated into the Ifla-CBPV and Dicistro mRT-PCR assays. The developed IAC-controlled mRT-PCR assays allow for simultaneous detection of mixed viral infections caused by six virus species commonly occurring in worldwide population of bees. The use of IAC during molecular detection enables monitoring of the assays correct performance.

**Supplementary Information:**

The online version contains supplementary material available at 10.1007/s11259-025-11012-3.

## **Introduction**

Honey bees are eusocial organisms living in colonies, which constitute a favorable environment for spreading different pathogens, including viruses, within the colony. Hitherto, over seventy virus species have been detected in bees, including deformed wing virus (DWV), sacbrood virus (SBV), acute bee paralysis virus (ABPV), Israeli acute paralysis virus (IAPV), black queen cell virus (BQCV) and chronic bee paralysis virus (CBPV) which are serious threats to the bees’ health (Aubert 2008; Beaurepaire et al. 2020). All life stages of honey bees are susceptible to viral infections (Chen et al. 2006). For instance, SBV and BQCV infect a honey bee brood, while DWV, ABPV, IAPV and CBPV cause infections mainly in adult bees (Ribiere et al. 2008). Although viral infections in bees are mainly characterized by an asymptomatic course, they still may have a negative impact not only on the individual bee, but also on the whole colony (Tentcheva et al. 2004; Aubert 2008; D’Alvise et al. 2019; Ullah et al. 2021). In conditions under which immune system of the insects are suppressed, symptoms of viral diseases may appear, which usually lead to bee’s death (Genersch et al. 2010; Pohorecka et al. 2011a, 2014; Francis et al. 2013; Budge et al. 2015). Because of the asymptomatic course of viral infections, their detection needs employment of methods characterized by a high diagnostic sensitivity and specificity (Aubert 2008). Currently, molecular methods such as PCR are mainly employed in diagnostics of bee viruses as well as in the epidemiological studies of bee viral diseases (Forgách et al. 2008; Genersch et al. 2010; Gauthier et al. 2011; Gregorc and Bakonyi 2012; Pohorecka et al. 2014; Shumkova et al. 2018). Their great advantage is the possibility of a simultaneous detection of several virus species in the tested sample, which significantly reduces the cost and time of sample analysis (De Miranda 2008). On the other hand, PCR-based methods utilize enzymes which are prone to different sample-derived inhibitory substances, which could result in obtaining false results due to reaction inhibition (De Miranda 2008). Therefore, when molecular assays are being used, a broad set of controls should be employed, of which the external or internal amplification controls (IAC) are of upmost importance (Hoorfar et al. 2004b; Schrader et al. 2012). IAC allows efficient monitoring of amplification of the target virus sequence present in the sample. It should be an integral part of any PCR-based diagnostic assay (Hoorfar et al. 2004b; Schrader et al. 2012). It is noteworthy that the reliable results can only be obtained when the method used was carefully optimized and validated, confirming its diagnostic performance.

In this study, we report a development and optimization of the Ifla-CBPV and Dicistro multiplex RT-PCR (mRT-PCR) assays containing the IAC for the detection of honey bee dicistroviruses (ABPV, IAPV, BQCV), iflaviruses (DWV-A, SBV) and CBPV. In the next phase of the study, the developed mRT-PCR assays will be validated according to WOAH recommendations.

## Materials and methods

### Viral RNA

RNA of iflaviruses (DWV-A, SBV), dicistroviruses (ABPV, IAPV, BQCV) and CBPV was isolated from samples of dead bees collected between 2010 and 2014 from Polish apiaries in which viral infections were reported. Mixtures of viral RNA were used for optimization of the developed Ifla-CBPV and Dicistro mRT-PCRs.

### Nucleotide sequences of bee viruses

The sequences of domestic and foreign strains of ifla- (DWV**-**A and B, SBV) and dicistroviruses (ABPV, IAPV, BQCV), as well as CBPV (Tables S1, S2, S3, S4, S5, S6 and S7) deposited in a GenBank, were used to refine the primers nucleotide sequences which were employed for development of Ifla-CBPV and Dicistro mRT-PCR assays. The sequences represented fragments or the whole genes of helicase (DWV), polymerase (BQCV and CBPV) and VP4 protein (ABPV). An intergenic region of IAPV genome and the 5’ end of SBV genome were also analyzed.

### Bacterial DNA

DNA of *Bordetella bronchiseptica* (ATCC 4617) and *Salmonella enterica subsp. enterica serovar Typhimurium* (ATCC 14028) was used to construct the IAC for the Ifla-CBPV and Dicistro mRT-PCR assays.

### Primers selection for mRT-PCR assays

Currently published set of PCR primers for DWV (Chen et al. 2005a), CBPV (Blanchard et al. 2009), BQCV (Blanchard et al. 2007), SBV (Grabensteiner et al. 2001), ABPV (Bakonyi et al. 2002) and IAPV (Cox-Foster et al. 2007) detection was chosen for a development of Ifla-CBPV and Dicistro mRT-PCRs. Based on the multiple sequence alignment of particular genome fragments of Polish and foreign virus strains (Tables S1, S2, S3, S4, S5, S6 and S7) using a MEGA 7.0 software (Kumar et al. 2016), all primers pairs, except for these primers designed for CBPV detection, were modified to increase their specificity and broad detection of viruses of particular species (Table 1). DWV primers were modified to allow a specific detection of DWV-A variants detected worldwide (Table S1, S2). Primers were synthesized by Genomed S.A. and stored at − 20 °C until use.

**Table 1:** The primers used in the Ifla-CBPV and Dicistro mRT-PCR assays. Nucleotide modifications in the primer sequences are denoted as Y (C or T), W (A or T), K (G or T), R (A or G) and written in italics

Assay	Starter name	Virus	Sequence (5’ → 3’)	Position in the genome		Product size (bp)	References
Ifla-CBPV mRT-PCR	DWV-sense	DWV-A	GCG*YW*TAGTGGAGGAAATGAAG	6259–6280	706		Chen et al. 2005a with the authors’ own modifications
	DWV-antisense		CGACAATTT*K*CGGACATCAATAAG	6941–6964			
	CBPV A3	CBPV	TCAGACACCGAATCTGATTATTG	1921–1943	570		Blanchard et al. 2009
	CBPV A2		ACTACTAGAAACTCGTCGCTTCG	2468–2490			
	SB 1f	SBV	ACCAACC*R*ATTCC*Y*CAGTAG	221–240	469		Grabensteineret al. 2001 with the authors’ own modifications
	SB 2r		CCTT*R*GAACT*Y*TGCTGTGTA	670–689			
Dicistro mRT-PCR	IAPV IGR F	IAPV	*Y*GATGAACAACGGAAGGTTT	6128–6147	767		Cox-Foster et al. 2007with the authors’ own modifications
	IAPV IGR R		ATCG*A*CT*R*AGGGGTTTGTTT	6875–6894			
	BQCV 3	BQCV	GGTG*Y*AA*R*TCTCTTCCTAG	5015–5033	606		Blanchard et al. 2007 with the authors’ own modifications
	BQCV 4		*CR*TA*R*CCT*R*AAAGGCCAAGAG	5600–5620			
	ABPV 1	ABPV	CATA*Y*TGGCGAGC*Y*ACTATG	8115–8134	398		Bakonyi et al. 2002 a with the authors’ own modifications
	ABPV 2		CCACTTCCA*Y*ACAACTAT*Y*G	8493–8512			

### Preparation of viral RNA mixtures

RNA of iflaviruses (DWV-A, SBV) and dicistroviruses (ABPV, IAPV, BQCV) as well as CBPV was isolated from dead bees collected from Polish apiaries in which viral infections were detected. Before preparation of viral RNA mixtures, RNA solutions of each virus species were prepared. Briefly, 10 individual worker bees were pooled, immersed in a liquid nitrogen and homogenized using a mortar and pestle. The obtained bee homogenate was used for RNA isolation using a Total RNA kit (A&A Biotechnology, Poland) according to the manufacturer’s instruction. RNA was eluted with 100 µl of nuclease-free water. The obtained RNA solutions contained from 1,000 to 100,000 PCRU of viral RNA (EURL 2013). Subsequently, individual RNA samples were combined to obtain RNA mixtures containing 10 PCRU of each virus species. RNA mixtures were stored at −80 °C until use.

### IAC construction for the Ifla-CBPV and Dicistro mRT-PCR

The IAC for the Ifla-CBPV mRT-PCR was constructed based on a DNA fragment of the InvA *S. typhimurium* gene, however for Dicistro assay, DNA of *B. bronchiseptica* flagellin gene was used. In order to obtain the 324 bp (Ifla-CBPV) or 204 bp (Dicistro) IAC products, two sets of the primer pairs (IAC F and R Ifla-CBPV; IAC F and R Dicistro) were designed, each composed of T7 polymerase promotor sequence, nucleotide sequences of the SBV or ABPV primers as well as primers used for *S. typhimurium* or *B. bronchiseptica* detection (Table 2).

**Table 2:** The primers used for IAC DNA constructs preparation. The sequences of *S. typhimurium* and *B. bronchiseptica* primers are in italics . The T7 polymerase promoter sequence (AATTCTAATACGACTCACTATAGGGAGAAGG) is present in IAC F Ifla-CBPV and Dicistro primers. Nucleotide modifications in the primer sequences are denoted as Y (C or T), W (A or T), K (G or T) and R (A or G)

Primer	Sequence (5’ → 3’)	Product size (bp)	Assay	References
IAC F Ifla-CBPV	AATTCTAATACGACTCACTATAGGGAGAAGGACCAACCRATTCCYCAGTAG*GTGAAATTATCGCCACGTTCGGGCAA*	324	Ifla-CBPV mRT-PCR	Grabensteiner et al. (2001) with the authors’ own modifications; Rahn et al. 1992
IAC R Ifla-CBPV	CCTTRGAACTYTGCTGTGTA*TCATCGCACCGTCAAAGGAACC*			
IAC F Dicistro	AATTCTAATACGACTCACTATAGGGAGAAGGCATAYTGGCGAGCYACTATG*CCCCCGCACATTTCCGAACTTC*	204	Dicistro mRT-PCR	Bakonyi et al. (2002) with the authors’ own modifications; Hozbor et al. 1999
IAC R Dicistro	CCACTTCCAYACAACTATYG*AGGCTCCCAAGAGAGAAAGGCTT*			

The oligonucleotides were synthesised at Genomed S.A. in Warsaw. Amplification of the relevant IAC product was carried out in 50 µl reaction volume containing: 1 x reaction buffer (Invitrogen, USA), 1.5 mM MgCl_2_, 200 µM of a mixture of dNTPs (Invitrogen, USA), 2 U of Platinum *Taq* DNA polymerase (Invitrogen, USA), 0.4 µM of each of the IAC F and R Ifla-CBPV primers or 0.8 µM of the IAC F and R Dicistro oligonucleotides (Table 2), 5 µl of *S. typhimurium* or *B. bronchiseptica* DNA and DNase- and RNase-free water to the desired volume. The following temperature-time profile was used: initial denaturation (94 °C, 2 min) followed by 35 cycles, each consisting of denaturation (94 °C, 45 s), primer annealing (71 °C for Ifla-CBPV or 55 °C for Dicistro at 45 s) and extension stage (72 °C, 45 s). The final extension step occurred at 72 °C for 5 min. The IACs DNA was purified and transcribed according to the manufacturer’s instructions using respectively a QIAquick^®^ Gel Extraction Kit (Qiagen, Germany) and a Riboprobe^®^ System-T7 kit (Promega, USA). IACs RNA was suspended in 30 µl of DNase- and RNase-free water. The concentration and purity of IACs RNA were determined with NanoPhotometer (Implen, Germany) by measuring UV light absorbance at 260 and 260/280 nm. IACs RNA was diluted to the concentration of 10 ng/µl and stored at − 80 °C until use.

### Development of the Ifla-CBPV and Dicistro mRT-PCRs

Viral RNA mixtures (5 µl) consisting of 10 PCRUs of respective virus species served as samples used for optimization of Ifla-CBPV and Dicistro mRT-PCRs. Reactions were conducted in two-step format using random hexamer primers and RevertAid First Strand cDNA Synthesis Kit (Thermo Scientific, USA) for reverse transcription (RT) step. Composition of RT mixture and time-temperature profile used followed the manufacturers’ recommendations, except for addition of IAC RNA to reaction mixture. For optimization of the IAC concentration, the relevant solutions of IAC RNA containing from 500 to 0.5 pg RNA/5 µl were added to each Ifla-CBPV or Dicistro RT reaction.

The particular PCRs were carried out using a Platinum *Taq* DNA polymerase (Invitrogen, USA) in the presence of 5 µl of viral cDNA. Briefly, the concentrations of the following components of the PCR mixtures were tested: specific primers for each virus species i.e. DWV-sense and antisense (0.05–0.5 µM), CBPV A2-A3 and ABPV 1–2 (0.1–0.5 µM), SB 1f-2r (0.25–0.7 µM), BQCV 3–4 and IAPV IGR F-R (0.1–1.0 µM), magnesium ions (from 1.5 to 3.5 mM), *Taq* DNA polymerase (from 2 to 4 U) and addition of bovine serum albumin (BSA) (from 5 to 20 µg). Initial denaturation of nucleic acids was carried out at 94 °C for 3 min followed by 35 cycles consisting of amplification at 94 °C for 60 s, primer annealing from 54 °C to 59 °C for 60 s, extension at 72 °C for 60 s, and a final extension step occurring at the same temperature for 10 min (Table S8). PCRs were carried out in a 50 µl reaction volume containing 1×PCR buffer (Invitrogen, USA), 200 µM of deoxyribonucleotide triphosphate (dNTP) mix (Invitrogen, USA), and molecular grade water to the required volume. Reactions were performed in triplicate by using a Mastercycler pro thermocycler (Eppendorf, Germany).

The correct performance of viral RNA isolation and amplification steps was monitored using the appropriate set of controls, i.e. a negative RNA extraction control (a sample free tube with all reagents used during viral RNA extraction), positive and negative reaction controls at molecular detection step (RT and PCR mixtures containing respectively a viral RNA or nuclease-free water as templates) and an environmental controls (open tubes left during the addition of RNA or cDNA sample).

PCR products were analyzed in 1.7% agarose gel containing SimplySafe dye (EURx, Poland). Electrophoresis was carried out in 1×TBE buffer at a constant voltage of 200 V (5 V/cm) for 40 min. The size of the amplicons obtained was compared to a DNA mass standard (GeneRuler 100 bp DNA Ladder, Thermo Fisher Scientific, Fermentas).

## Results

### Construction and optimization of IAC RNA concentration in the assay

DNA of *S. typhimurium* and *B. bronchiseptica* was used to construct the IACs. The transcription reaction of IACs DNA generated IACs RNA for which specific products of 324 bp (Ifla-CBPV assay) or 204 bp (Dicistro assay) were observed in mRT-PCR assays.

**Fig. 1:**
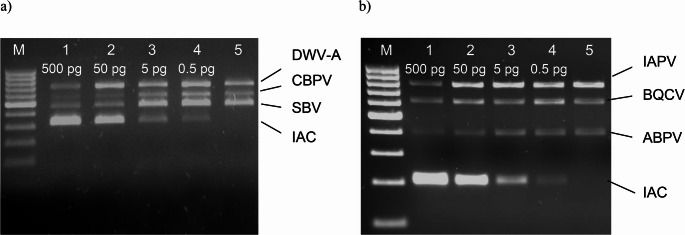
Results of optimization of IAC concentration in the Ifla-CBPV (**a**) and Dicistro (**b**) mRT-PCR assays. Lines: M – DNA marker (100 bp DNA Ladder); 1–4 - amplification of viral RNA templates in the presence of IAC at concentrations ranging from 500 to 0.5 pg; 5 - amplification of viral RNA templates without IAC

To obtain the highest assays sensitivity and to avoid interference with the amplified target sequences, the concentration of IACs RNA in the RT reaction was determined. Both IACs produced a visible signal when the Ifla-CBPV and Dicistro mRT-PCR reaction contained from 0,5 pg to 500 pg of IAC RNA. IACs at concentrations above 5 pg decreased the efficiency of viral templates amplification, but at concentrations of 5 pg or below they had no adverse effect on amplification of the viral templates, and therefore this IACs concentration was observed to be optimal (Fig. 1).

The RT reaction of Ifla-CBPV and Dicistro mRT-PCR assays was carried out according to the manufacturer’s instructions, taking into account the addition of 5 pg of IAC RNA in the reaction mixture (Tables 3 and 4).

**Table 3:** The composition of RT mixture for the Ifla-CBPV and Dicistro mRT-PCR assays

Ingredient	Volume [µl]	Concentration
Nuclease-free water	1	-
5x Reaction Buffer	4	1x
dNTPs	2	1 mM
Random hexamer	1	5 µM
RiboLock RNase inhibitor	1	20 U
RevertAid Reverse Transcriptase	1	200 U
RNA of Ifla-CBPV or Dicistro IAC	5	5 pg
RNA sample	5	-
Total	20	-

**Table 4:** Temperature-time profile of the RT step for the Ifla-CBPV and Dicistro mRT-PCR assays

Stage	Temperature (°C)	Time (min)
Initial incubation	25	5
cDNA synthesis	42	60
Final incubation	70	5

### Primers selection

The selected Ifla-CBPV and Dicistro mRT-PCR primers annealed within the conserved genome fragments of Polish and foreign strains of ifla-, dicistroviruses and CBPV available in GenBank. The primers allowed amplification of gene fragments of particular virus species ranging from 398 bp to 767 bp (Table 1). The primer sequences for ABPV, SBV, BQCV and IAPV were modified having regard to presence in their sequences of point mutations occurring in homologous genome fragments of analyzed domestic and foreign virus strains. The modification of DWV-sense and DWV-antisense oligonucleotides also increased their specificity, ensuring specific detection of DWV-A strains. The sequences of the CBPV A2-A3 primers, which anneal within the CBPV polymerase gene sequence (Blanchard et al. 2009), were 100% identical to the homologous gene sequences of both domestic and foreign virus strains.

### Optimization of the assays

In order to determine the optimal composition of the reaction mixtures of the Ifla-CBPV and Dicistro mRT-PCRs, as well as the amplification conditions, the effects of different concentrations of *Taq* polymerase, magnesium ions and supplementation of the reaction mixtures with bovine serum albumin on reaction efficiency were assessed. The concentration of primers and the temperature of their annealing to the cDNA template were also optimized (Table S8).

The amplification efficiency of all tested templates in reaction mixtures containing 2 to 4 U *Taq* polymerase/reaction in both the Ifla-CBPV and Dicistro mRT-PCR assay was comparable. However, based on the results of the spectrophotometric evaluation of the PCR products’ concentration in the reaction mixture, it was shown that increasing the amount of *Taq* polymerase from 2 to 2.5 U/reaction resulted in an increase in amplification efficiency of 21% for the Ifla-CBPV assay and 28% for Dicistro assay. Polymerase concentration higher than 2.5 U did not improve the amplification efficiency of the viral templates, regardless of the mRT-PCR assay used. Therefore, the 2.5 U *Taq* polymerase/reaction concentration was considered optimal. When 1.5 to 3.5 mM magnesium ions were added to the Ifla-CBPV and Dicistro mRT-PCR reaction mixtures, differences in reaction efficiency were also observed only in the spectrophotometric measurement. Increasing the magnesium ion concentration from 1.5 to 2 mM in the Dicistro mRT-PCR assay led to a 35% increase in the amount of PCR products, whereas higher concentrations decreased reaction efficiency. In contrast, in the Ifla-CBPV mRT-PCR assay, increasing the amount of magnesium ions from 1.5 to 3.5 mM was not accompanied by an increase in amplification efficiency. The optimal magnesium ion concentration was considered to be 1.5 mM for the Ifla-CBPV mRT-PCR assay and 2 mM for the Dicistro mRT-PCR. The amplification efficiency of reaction mixtures without BSA and with the addition of BSA in the range of 5 to 20 µg was also compared. Amplification of virus templates in both the Ifla-CBPV and Dicistro mRT-PCR assay was most efficient at a concentration of 20 µg BSA/reaction.

In the range of primer annealing temperatures assessed (54–59 °C), mRT-PCR assays produced specific amplicons for all viral templates tested, i.e. IAPV, BQCV, ABPV, DWV-A, CBPV and SBV. In the Ifla-CBPV mRT-PCR assay, a similar amplification signal intensity was observed, irrespective of the temperature used, therefore, the selection of the optimal temperature was based on a spectrophotometric evaluation of the PCR product concentration in the individual reaction mixtures. The highest amplification efficiency was obtained at 56 °C, which was assumed to be the optimum temperature. For the Dicistro mRT-PCR assay, the highest reaction efficiency was also observed at 56 °C.

**Fig. 2:**
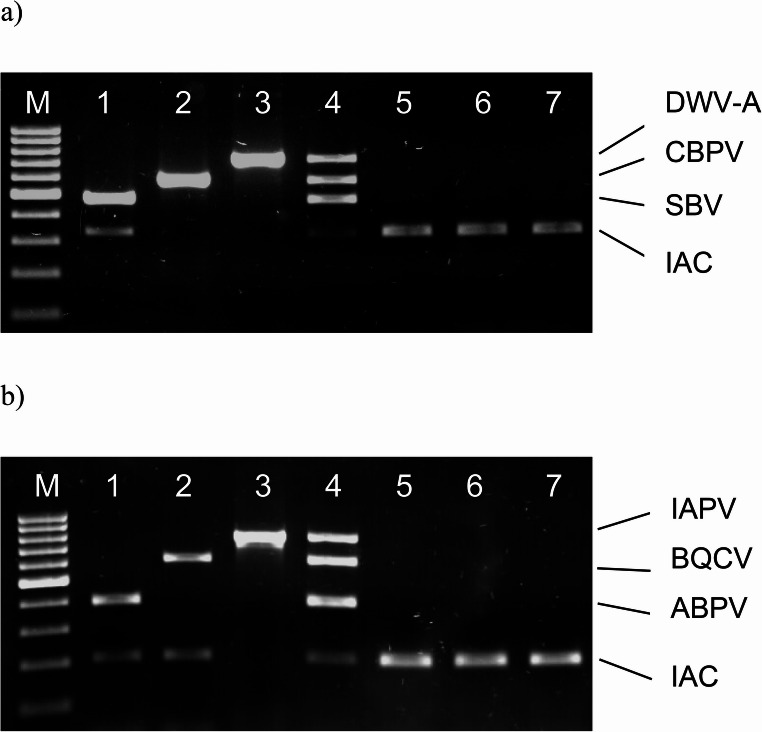
Results of optimization of the Ifla-CBPV (**a**) and Dicistro (**b**) mRT-PCR assays. a, b: line M – DNA marker (100 bp DNA Ladder); a: amplification of SBV (line 1), CBPV (line 2) and DWV-A (line 3) template; b: amplification of ABPV (line 1), BQCV (line 2) and IAPV (line 3) template; a, b: line 4 - amplification of viral RNA sample containing 10 PCRUs of each virus template: SBV, CBPV and DWV-A (Ifla-CBPV assay) or ABPV, BQCV and IAPV (Dicistro assay); line 5 – negative control of RNA extraction; line 6 – negative control of RT-PCR mixture; line 7 – environmental RT-PCR control

The optimized Ifla-CBPV and Dicistro mRT-PCR assays were carried out in a 50 µl reaction volume containing 1 × reaction buffer, 200 µM dNTPs, 1,5 (Ifla-CBPV) or 2 mM (Dicistro) MgCl_2_, 0.2 µM of DWV-sense/antisense, 0.7 µM of SB 1f-2r and 0.15 µM of CBPV A2-A3 primers (Ifla-CBPV mRT-PCR) or 0.5 µM of IAPV IGR F-R, 0.9 µM of BQCV 3–4 and 0.4 µM of ABPV 1–2 primers (Dicistro mRT-PCR), 2,5 U Platinum *Taq* DNA polymerase, 20 µg BSA and 5 µl of cDNA sample (Tables 5 and 6). The following optimal temperature profile was used: initial denaturation at 94 °C for 3 min and 35 cycles consisting of denaturation at 94 °C for 60 s, annealing at 56 °C for 60 s, elongation at 72 °C for 60 s and a final elongation step at 72 °C for 10 min (Table 7). The developed mRT-PCR assays were able to successfully detect all tested viruses. The controls used (including the IAC) confirmed the correct assays performance (Fig. 2).

**Table 5:** The composition of the optimized PCR mixture of the Ifla-CBPV mRT-PCR assay

Ingredient	Volume [µl]	Concentration
DNase-free water	25.75	-
10 x PCR buffer (without MgCl_2_)	5	1x
MgCl_2_	1.5	1.5 mM
dNTPs	1	200 µM
DWV-sense	1	0.2 µM
DWV-antisense	1	0.2 µM
CBPV A3	0.75	0.15 µM
CBPV A2	0.75	0.15 µM
SB 1f	3.5	0.7 µM
SB 2r	3.5	0.7 µM
BSA	1	20 µg
Platinum *Taq* DNA polymerase	0.25	2.5 U
cDNA sample	5	-
Total	50	-

**Table 6:** The composition of the optimized PCR mixture of the Dicistro mRT-PCR assay

Ingredient	Volume [µl]	Concentration
DNase-free water	17.75	-
10 x PCR buffer (without MgCl_2_)	5	1x
MgCl_2_	2	2.0 mM
dNTPs	1	200 µM
IAPV IGR F	2.5	0.5 µM
IAPV IGR R	2.5	0.5 µM
BQCV 3	4.5	0.9 µM
BQCV 4	4.5	0.9 µM
ABPV 1	2	0.4 µM
ABPV 2	2	0.4 µM
BSA	1	20 µg
Platinum *Taq* DNA polymerase	0.25	2.5 U
cDNA sample	5	-
Total	50	-

**Table 7:** Temperature-time profile of the PCR step for the Ifla-CBPV and Dicistro mRT-PCR assays

Stage	Temperature (°C)	Time (min)	Number of cycles
Initial denaturation	94	3	-
Denaturation	94	1	35
Annealing	56	1	
Elongation	72	1	
Final elongation	72	10	-

## Discussion

Viral infections occur in honey bees worldwide (Beaurepaire et al. 2020). The global spread of bee viruses has been facilitated by the development of apiculture, international trade and transport of bees as well as drone semen (Moritz et al. 2005; Mutinelli 2011). Viral infections easily spread within a bee colony causing immunosuppression, increasing bee mortality or extinction of the whole colonies (Berenyi et al. 2006; Berthoud et al. 2010; Genersch et al. 2010; Pohorecka et al. 2011a, 2014; Francis et al. 2013; Budge et al. 2015). Currently, there are no treatment options or vaccines available to combat viral diseases of bees. Consequently, the transmission of viral infections within bee colonies can only be mitigated through the implementation of the hygienic and breeding measures. Therefore, to efficiently control the occurrence of viral diseases in bees, there is a need for a broad employment of laboratory diagnostics of bee viruses (Aubert 2008). Initially, for the detection of viral infections in bees, the serological methods such as AGID and ELISA have been used (Bailey and Gibbs 1964; Anderson and Gibbs 1988; Topolska et al. 1995, Shen et al. 2005a, b; Topolska 2008). However, due to a low sensitivity of these assays or a lack of commercially available diagnostic reagents, they are not currently used (Ribiere et al. 2010). Nowadays, PCR-based methods are commonly used for the detection of bee viruses (Stoltz et al. 1995; Chen et al. 2004, 2005a; Meeus et al. 2010; Pohorecka et al. 2011a, b, 2014; Kevil et al. 2017; Barroso-Arevalo et al. 2019a; Cagirgan and Yazici 2020). Beside a speed of the analysis, they allow for a simultaneous detection of the mixed infections caused by several virus species (Chen et al. 2004; Teixeira et al. 2008; Sguazza et al. 2013; Cagirgan and Yazici 2020). However, currently published RT-PCR assays for detection of bee viruses usually did not employ the amplification controls in the analytical process or have not been validated to confirm their diagnostic suitability (Chen et al. 2004, 2005b; Topley et al. 2005; Yue et al. 2006; Grabensteiner et al. 2007; Teixeira et al. 2008; Gregorc and Bakonyi 2012; Reynaldi et al. 2013; Sguazza et al. 2013; Gregorc and Smodiš Škerl 2015; Cagirgan and Yazici 2020).

In the present study, the development of Ifla-CBPV and Dicistro mRT-PCR assays containing the IAC was described. The tests allow for a simultaneous detection of dicistro- (ABPV, IAPV, BQCV), iflaviruses (DWV-A, SBV) and CBPV in bees. The assays amplify the conservative regions of the viral genomic RNA which ensures their high sensitivity and specificity. Similar diagnostic approach has been presented by Sguazza et al. (2013) for the detection of ifla- (DWV, SBV), dicistroviruses (ABPV, IAPV and BQCV) and CBPV. A high specificity of the method was obtained by design of the assay’s primers within the conservative regions of the viral genomes. Other mRT-PCR assay for simultaneous detection of BQCV, ABPV, and DWV has been also previously described (Teixeira et al. 2008). In this assay, the full-genome nucleotide sequences of particular virus species were used for assay design. This could have a positive impact on its sensitivity and specificity. In the case of other assay used for detection of BQCV, ABPV and SBV in brood samples collected from asymptomatically infected bee colonies, a simple modification of the primer sequences by incorporation of a deoxyinosine nucleoside increased mRT-PCR sensitivity (Topley et al. 2005). The Ifla-CBPV and Dicistro mRT-PCRs developed in this study were subjected to optimization which encompassed the following parameters of the assays: determination of the reaction temperature profile as well as concentration of primers, magnesium ions and *Taq* polymerase in the PCR mixture. The effect of reaction mixture supplementation with BSA, which was found to have a beneficial effect on the amplification efficiency of viral templates, was also evaluated. This observation is consistent with results of other studies in which BSA supplementation reduced the negative impact of sample-derived organic compounds on amplification efficiency (Henegariu et al. 1997; Hedman and Rådström 2013). It has been also shown that albumin protects *Taq* polymerase from tissue proteases (Hedman and Rådström 2013). Likewise, a higher concentration of polymerase and magnesium ions in the PCR mixture improved overall reaction efficiency of the Ifla-CBPV and Dicistro mRT-PCRs and these results are consistent with previous findings (Chamberlain et al. 1988; Henegariu et al. 1997).

Although PCR-based methods offer a high diagnostic sensitivity and specificity, they can also deliver false results as they are prone to inhibition of enzymatic reactions by substances present in the tested sample (Belák and Ballagi-Pordány 1993; Valentine-Thon 2002; Hoorfar et al. 2004a). Therefore, a set of suitable controls, including internal or external amplification controls should be used to monitor the amplification course during molecular analyses (Hoorfar et al. 2004b; Maaroufi et al. 2006). The newly developed Ifla-CBPV and Dicistro mRT-PCRs contain the IAC that is amplified in the same reaction tube as the sample matrix using a common primer pair. This approach allows to fully control the amplification process, however it requires optimization of the IAC concentration in the reaction mixture to avoid side effects related to lower amplification efficiency of the virus template and decreased assay sensitivity due to a high IAC content (Rosenstraus et al. 1998). In addition, taking into account a kinetics of molecular reaction as well as the observed competition between amplified matrices for the reaction components, the designed IAC should have a larger size than the amplified fragment of the virus sequence (Hoorfar et al. 2004b). In this study, the amplified IAC fragments were shorter than the target virus sequences, nevertheless the sensitivity of the developed mRT-PCRs has not been negatively affected. This observation has been also confirmed in other studies (Brightwell et al. 1998; Abdulmawjood et al. 2002). It is noteworthy that, in the currently published PCR protocols for detection of bee viruses, a sample-derived constitutive genes of the bees, e.g. 18 S rRNA mitochondrial sequences, β-actin gene, have occasionally been used as amplification controls (Shen et al. 2005a, b; Ward et al. 2007; Delaney et al. 2011; Antunez et al. 2012; Yoo et al. 2012; Kevill et al. 2017). However, the use of the constitutive genes as controls did not fully reflect amplification conditions of the target virus sequences (Schrader et al. 2012). On the other hand, the inhibitory effect of the sample matrix on the PCR detecting honey bee dicistroviruses (ABPV, IAPV, KBV) and DWV has been controlled by amplification of the MS2 bacteriophage RNA (Meeus et al. 2010). Other diagnostic approach relates to spiking of the tested sample with non-target virus species, e.g. tobacco mosaic virus, before analysis of the sample began. Notably, this particular approach should permit not only a monitoring of the sample inhibitors, but also the extraction efficiency of viral RNA and cDNA synthesis (Tentcheva et al. 2004, 2006; Gauthier et al. 2007, 2011). However, in contrast to the IAC, other controls require the use of an additional primer pair in the reaction, which can decrease the amplification efficiency of the virus template (Hoorfar et al. 2004b).

The main limitations of the current study are: (i) the developed assays were only employed for detection of viral infections in adult bees without their testing for virus detection in other developmental stages of bees which are also susceptible to infection, (ii) the Ifla-CBPV mRT-PCR was designed to detect only DWV-A strains. However, a higher prevalence of DWV-A variants has been observed in colonies exhibiting increased bee mortality, in contrast to DWV-B (VDV 1) variants, which are primarily detected in healthy colonies (Mordecai et al. 2016; Kevill et al. 2017, 2019; Barroso-Arevalo et al. 2019b; Simenc et al. 2021; Oz et al. 2023). Although the Ifla-CBPV and Dicistro mRT-PCRs have been developed for detection of viral infections in adult bees (workers), they should still allow virus detection in other developmental stages (eggs, larvae, pupae) if a suitable method for extraction of the viral RNA will be employed. The developed Ifla-CBPV and Dicistro mRT-PCRs allow for the simultaneous detection of six virus species commonly occurring in the population of bees. The incorporation of IAC into the assays allows for monitoring of their correct performance and avoidance of generation of false-negative results. Subsequent validation of the assays will provide data on their analytical performance characteristics and fully confirm their diagnostic suitability.

## Supplementary Information

Below is the link to the electronic supplementary material.

Supplementary Material 1 (PDF. 379 KB)

## Data Availability

The sequences generated during this study were deposited in the NCBI GenBank (https://www.ncbi.nlm.nih.gov/genbank/). Sequences can be accessed by the following accession numbers: OR513772 - OR513792 and OR576827 - OR576831. Detailed information on DWV-A and B (VDV 1), CBPV, SBV, ABPV, BQCV and IAPV strains used to develop the mRT-PCR assays as well as the optimized parameters of mRT-PCR assays are provided in the Supplementary Tables (Tables S1, S2, S3, S4, S5, S6, S7 and S8).
