# Supplementary material for: Development of multiplex RT-PCR assays containing an internal amplification control for the detection of dicistro-, iflaviruses and CBPV in honey bees. Part 1 - assays design and optimization
Source: Vet Res Commun. 2026 Jan 15;50(2):106. doi: 10.1007/s11259-025-11012-3 (PMC12808220; doi:10.1007/s11259-025-11012-3)
Supplement: Supplementary file 1 — Supplementary Material 1 (PDF. 379 KB) [file 11259_2025_11012_MOESM1_ESM.pdf]

Development of multiplex RT-PCR assays containing an internal amplification control for the detection of dicistro-, iflaviruses and CBPV in honey bees. Part 1- assays design and optimization

Veterinary Research Communications

Dagmara Zdańska[1*], Artur Rzeżutka[2], Krystyna Pohorecka[3]

[1] Department of Parasitology and Invasive Diseases, Bee Diseases and Aquatic Animal Diseases, National Veterinary Research Institute, Al. Partyzantów 57, 24-100 Puławy, Poland

*Corresponding Author: dagmara.zdanska@piwet.pulawy.pl

Table S1 Detailed information on strains of bee iflaviruses, dicistroviruses and CBPV detected in honey bee populations in Poland that were used for molecular studies

Virus	Fragment of virus gene/genome	Voivodeship	Year of detection	GenBank no.
DWV-A	helicase	pomorskie	2013	OR513788
		wielkopolskie	2014	OR513787
		mazowieckie		OR513785
		lubuskie		OR513786
CBPV	polymerase	wielkopolskie	2014	OR513784
		mazowieckie		OR513783
		kujawsko-pomorskie		OR513782
SBV	the 5' end of the genome	mazowieckie	2014	OR513791
		lubelskie		OR513790
		łódzkie		OR513792
		lubuskie		OR513789
ABPV	VP4 protein	opolskie	2009	OR513772
		zachodniopomorskie	2011	OR513773
		lubuskie	2012	OR513774
		lubelskie	2014	OR513775
		warmińsko-mazurskie		OR513776
BQCV	polymerase	opolskie	2011	OR513777
		zachodniopomorskie		OR513778
		mazowieckie	2014	OR513779
		lubelskie		OR513780
		warmińsko-mazurskie		OR513781
IAPV	intergenic region	śląskie	2008	OR576827
				OR576828
		ND	2009	OR576829
		małopolskie	2010	OR576830
		ND	2011	OR576831

ND – no data

Table S2 DWV-**A** and DWV-B (VDV 1) strains used to develop the Ifla-CBPV mRT-PCR assay. DWV-B strains are marked with an asterisk

Name/designation of the strain	Host/ stage of development	Country of origin	Year of detection	GenBank no.
Poland 1 - 5	ND/worker bee	Poland	2003	DQ224287-91
Germany 1 - 3	ND/worker bee	Germany	2003	DQ224292-94
Austria 2 - 9	ND/worker bee	Austria	2003	DQ224279-86
Austria 1414	honey bee/pupa		2014	KU847397
Hungary 1 - 3	ND/worker bee	Hungary	2003	DQ224295-97
Slovenia 1 - 4	ND/worker bee	Slovenia	2003	DQ224300-03
85-DWV	honey bee/ worker bee	France	2013	KX373899
A03, A04, A07, F13AL022			2013-2014	KX373903-06
F13CO06, F13CO05			2013	KX373914-15
F14PA149, F13PA010, F13PA028, F14PA038, F14PA040, F14PA043, F14PA047, F14PA050, F14PA052, F14PR097, F14PR099, F14PR102			2013-2014	KX373918-29
F14SA064, F14SA066			2014	KX373935-36
DWV	ND		ND	AY224602
DWV_MR, DWV_MS	honey bee/ worker bee	Sweden	2009	MH267695-96
Warwick-2009	honey bee/drone larva	the United Kingdom	2009	GU109335
DWV_Ox	honey bee/ worker bee		2010	KC786223
DWV	honey bee/ worker bee	Italy	2000	AJ489744
IT13AR029, IT13AR030			2014	KX373908-09
Nepal 1 - 2	ND/worker bee	Nepal	2003	DQ224304-05
DWV-JL1	honey bee/ND	China	2011	KP096414
GW, YD, NW, ES, CW, GN, DY, CA, GS1, HS1, MY, GS2	honey bee/ worker bee	South Korea	2012	JX878288-99
GR, SJ, HS2, Korea-1, 2				JX878301-05
U.A.E.-1, U.A.E.-2	ND /worker bee	United Arab Emirates	2003	DQ224308-09
Sri Lanka 1 - 2	ND/worker bee	Sri Lanka	2003	DQ224306-07
PA	honey bee/ worker bee	the USA	2000	AY292384
Canada 1 - 2	ND/worker bee	Canada	2003	DQ224310-11
AR14BB125, AR14BB126, AR14BB128, AR14LP112	honey bee/ worker bee	Argentina	2014	KX373910-12, KX373917
Chilensis A1	ND	Chile	2011	JQ413340
VDV-1 Ox*	honey bee	the United Kingdom	2010	KC786222
VDV-1*	*V. destructor*	Netherlands	ND	AY251269
VDV-1 Zriffin*	honey bee	Israel	2008	JF440525

ND – no data

Table S3 CBPV strains used to develop the Ifla-CBPV mRT-PCR assay

Name/designation of the strain	Country of origin	Year of detection	GenBank no.
1 M, 3 NZ, 4 RZ, 1 W	Poland	2006	FJ345318-21
A-79P	France	ND	EU122229
B-441			EU122231
274, 198, 596, 351, 363		2007	FJ345312-16
BE 104, AT34, BE 78	Austria	2003-2006	FJ345306-08
H4 300	Hungary	2005	FJ345317
L-4	Denmark	2007	FJ345310
23	Belgium	2006	FJ345309
R1-C6, R2-C102, R3-C10	Switzerland	2004	FJ345322-24
B4 V	Spain	2006	FJ345311
Wyy4	China	2011	KJ599572
8-C, 6-M, 13-F	Uruguay	2006	FJ345325-27

ND – no data

Table S4 SBV strains used to develop the Ifla-CBPV mRT-PCR assay

Name/designation of the strain	Host/ stage of development	Origin	Year of detection	GenBank no.
Brno	honey bee/ND	the Czech Republic	2014	KY273489
Rothamstead	honey bee/bee larva	the United Kingdom	ND	AF092924
SBV_MR, SBV_MS	honey bee/worker bee	Sweden	2009	MH267697-98
SBV	honey bee/bee larva	China	ND	AF469603
CSBV LN/China/2009	honey bee/bee larva	China	2009	HM237361
CSBV-FZ	eastern honey bee/ND	China	2012	KM495267
SXnor1	eastern honey bee/ND	China	2012	KJ000692
BJ 2012	eastern honey bee/bee larva	China	2012	KF960044
Korean	eastern honey bee/ND	South Korea	2010	HQ322114
AmSBV-Kor21, AmSBV-Kor19	honey bee/bee larva	South Korea	2011	JQ390591-92
AmSBV-Kor1	honey bee/worker bee	South Korea	2014	KP296800
AmSBV-Kor2	honey bee/worker bee	South Korea	2014	KP296801
AcSBV-Kor3	eastern honey bee/worker bee	South Korea	2014	KP296802
AcSBV-Kor4	eastern honey bee/worker bee	South Korea	2014	KP296803
II-2, K1A, K5B, K3A, S2, II-9	eastern honey bee/ND	India	2012	JX270795-800
II10	eastern honey bee/bee larva	India	2012	JX194121
SBM2	eastern honey bee/bee larva	Vietnam	2004	KC007374
LDst, HYnor	eastern honey bee/bee larva	Vietnam	2013	KJ959613-14
AcSBV-Viet1, AcSBV-Viet2	eastern honey bee/bee larva	Vietnam	2013	KM884990-91
AcSBV-Viet3	eastern honey bee/worker bee	Vietnam	2013	KM884992
AmSBV-Viet4	honey bee/bee larva	Vietnam	2013	KM884993
AcSBV-Viet5	eastern honey bee/worker bee	Vietnam	2013	KM884994
AmSBV-Viet6	honey bee/bee larva	Vietnam	2013	KM884995
SBV_WA2, SBV_WA1, SBV_VN3, SBV_VN2, SBV_VN1, SBV_TAS, SBV_SA, SBV_QLD, SBV_NT	honey bee/worker bee	Australia	2013-2014	KY465671-79

ND – no data

Table S5 ABPV strains used to develop the Dicistro mRT-PCR assay

Name/designation of the strain	Origin	Year of detection	GenBank no.
Poland 1	Poland	ND	AF486073
Poland 2 - 3			AY053370-71
Poland 4			AY053376
ABPV	the United Kingdom	ND	AF150629
Rothamsted			AF126050
Germany 1 - 2	Germany	ND	AY053367-68
Austria 1	Austria	ND	AY053366
Hungary 1	Hungary	ND	AF486072
Hungary 2 - 13			AY053372, AY053374, AY053375, AY053377-85

ND – no data

Table S6 BQCV strains used to develop the Dicistro mRT-PCR assay

Name/designation of the strain	Stage of development of honey bee	Origin	Year of detection	GenBank no.
Poland 4 - 6	queen pupa/larva	Poland	ND	EF517519-21
Hungary -10	ND	Hungary	ND	EF517515
Jl1	ND	China	2011	KP119603
AY	worker bee	South Korea	ND	JX149531
South African	drone pupa	the Republic of South Africa	ND	AF183905

ND – no data

Table S7 IAPV strains used to develop the Dicistro mRT-PCR assay

Name/designation of the strain	Origin	Year of detection	GenBank no.
4782IAPV	Spain	2010	JX045857
IAPV	Israel	ND	EF219380
IS1- Israel-2007		2007	EU436455
Wus-China-2008	China	2008	HQ897161
Korea 1, 3	South Korea	2012	KC690268, KC690270
IF31, IF55, IF56		ND	EU375539-41
IAPV	the USA	2003	EU218534
DVE31-OP3-PA-USA-2007		2007	EU436423
IAPV		ND	EU224279-80
OZ6-Australia-2007	Australia	2007	EU436456

ND – no data

Table S8 Tested parameters of the Ifla-CBPV and Dicistro mRT-PCR assays

Ingredient	Tested range (final concentration or amount)	
	Ifla-CBPV mRT-PCR	Dicistro mRT-PCR
DWV-sense i antisense	0.05 μM, 0.1 μM, 0.2 μM, 0.25 μM, 0.3 μM, 0.4 μM, 0.5 μM	-
SB 1f-2r	0.25 μM, 0.5 μM, 0.6 μM, 0.7 μM	-
CBPV A2-A3	0.1 μM, 0.15 μM, 0.2 μM, 0.25 μM, 0.3 μM, 0.4 μM, 0.5 μM	-
ABPV 1-2	-	0.1 μM, 0.2 μM, 0.3 μM, 0.4 μM, 0.5 μM
BQCV 3-4	-	0.1 μM, 0.2 μM, 0.3 μM, 0.4 μM, 0.5 μM, 0.6 μM, 0.7 μM, 0.8 μM, 0.9 μM, 1.0 μM
IAPV IGR F-R	-	
Magnesium ions	1.5 mM, 2.0 mM, 2.5 mM, 3.0 mM, 3.5 mM	
Taq polymerase	2 U, 2.5 U, 3 U, 3.5 U, 4 U	
BSA	5 μg, 10 μg, 15 μg, 20 μg	
IAC RNA	500 pg, 50 pg, 5 pg, 0.5 pg	
	Temperature	
Reaction primers (annealing temp.)	54.1°C, 55.0°C, 56.0°C, 57.1°C, 58.1°C, 59.1°C	
